# Genome-wide identification and expression analysis of PERK genes in peanut and revelation of bidirectional immune regulatory function

**DOI:** 10.3389/fpls.2025.1745895

**Published:** 2026-01-20

**Authors:** Jinfeng Peng, Wei Liu, Jian Yu, Danyang Fu, Yaya Sun, Xiangrong Zheng, Yuanyuan Chen, Fucai Xia, Jiajia Chen

**Affiliations:** 1 College of Landscape Architecture, Jiangsu Vocational College of Agriculture and Forestry, Zhenjiang, China; 2 College of Forestry, Beihua University, Jilin, China; 3 College of Forestry, Henan Agricultural University, Zhengzhou, Henan, China

**Keywords:** genestructure, genome-wide identification, PERK genes, phylogenetic analysis, plant innate immunity, tissue-specific expression

## Abstract

**Introduction:**

Proline-rich extensin-like receptor kinases (PERKs) represent a distinct subclass of plant receptor-like kinases (RLKs) ubiquitous in plants. While characterized in several species, a comprehensive analysis of the PERK gene family in cultivated peanut (*Arachis hypogaea* L.) remains limited.

**Methods:**

A genome-wide identification and systematic characterization of the PERK gene family in peanut was conducted. Evolutionary analysis was performed via phylogenetics and motif identification. Gene structures and promoter *cis*-elements were analyzed in silico. Expression profiles were assessed across tissues and under abiotic stresses. Functional validation of selected genes in plant innate immunity was performed.

**Results:**

Twenty-three PERK genes (*PERK1-PERK23*) were identified, unevenly distributed across 12 chromosomes (highest density on chromosome 5). Phylogenetic analysis with Arabidopsis PERKs classified them into three subgroups (I-III), with Subgroup II predominantly containing peanut members. All genes contain introns and share conserved motifs. Promoter analysis revealed stress-responsive elements, including light-responsive (all genes), MeJA-responsive (18 genes), and ABA-responsive (16 genes) elements. Expression profiling showed constitutive expression for 11 genes, ubiquitous high expression of PERK6/PERK20, and root/nodule-specific expression of *PERK13/PERK14*. Under abiotic stress, 12, 9, and 6 genes responded to low temperature, drought, and ABA, respectively. Functionally, *PERK4, PERK12*, and *PERK15* significantly suppressed plant innate immunity, whereas PERK8 enhanced it.

**Discussion:**

This study provides the first genome-wide analysis of the PERK family in peanut, revealing its evolutionary features and expression patterns. Crucially, functional characterization demonstrates that peanut PERKs can bidirectionally modulate plant innate immunity, with members acting as either negative or positive regulators. This discovery of their immune regulatory functions offers novel molecular targets for stress-resistance breeding in legume crops.

## 1 Introduction

Receptor kinases represent expansive gene families ubiquitously present across diverse plant species, including model systems such as *Oryza sativa* and *Arabidopsis thaliana*. The Arabidopsis genome encodes approximately 600 receptor kinase members, with functional homologs identified and characterized in approximately 20 plant species (Shiu and Bleecker, 2001a; Morris and Walker, 2003; Shiu et al., 2004). While the precise biological roles of most receptor kinases remain to be fully elucidated, they are established as pivotal regulators in diverse physiological processes, encompassing signal transduction pathways, environmental stress adaptation, hormone signaling cascades, plant growth and development, self-incompatibility mechanisms, cell differentiation programs, as well as symbiotic interactions and pathogen recognition (Shiu and Bleecker, 2001b; Diévart and Clark, 2004). The extracellular domains of receptor kinases exhibit specificity for binding diverse ligands, including steroids, peptides, carbohydrates, and cell wall components. Signal transduction across the plasma membrane is facilitated through conserved signaling complexes shared among eukaryotic cells, with the evolutionary origins of these receptors potentially traceable to the early emergence of multicellular organisms (Shiu and Bleecker, 2001a, b).

Receptor kinases are systematically classified into distinct categories based on characteristic extracellular domain motifs (Shiu and Bleecker, 2001b, 2003). Leucine-rich repeat (LRR) receptor kinases, for instance, constitute the largest class in Arabidopsis and include canonical brassinosteroid (BR) sensors such as BRI1 and BAK1 (Li and Chory, 1997; Li et al., 2002; Nam and Li, 2002). These kinases are indispensable for plant immunity; for example, LRR-containing FLS2 recognizes the conserved bacterial flagellin peptide flg22, triggering early immune defense responses such as ROS burst (Bauer et al., 2001; Zipfel et al., 2004). Functional redundancy, often arising from gene duplication events, is a common feature across different receptor kinase classes (Champion et al., 2004), exemplified by functionally overlapping CLV1 and ERECTA receptor kinases (Diévart et al., 2003; Shpak et al., 2003, 2004). *PERK*s constitute another significant subclass within the broader receptor kinase superfamily. The Arabidopsis *PERK* family exhibits its highest sequence similarity to Brassica napus *PERK1*, which is rapidly induced in response to wounding and in the presence of the pathogen *Sclerotinia sclerotiorum* (Silva and Goring, 2002). Fifteen *PERK* members have been annotated within the Arabidopsis genome, although their detailed biological functions remain largely unexplored (Silva and Goring, 2002; Nakhamchik et al., 2004). Arabidopsis *PERK1* localizes to the plasma membrane, and its expression is rapidly induced by mechanical wounding (Silva and Goring, 2002). Furthermore, *PERK4* was identified as a novel regulator of Ca²^+^signaling, mediating early events in ABA-responsive root tip growth inhibition (Bai et al., 2009a, b). Recent data showed that PERK expression is also regulated in response to environmental cues. *AtPERK13* is induced under phosphate, nitrogen and iron deprivations (Xue et al., 2021). PERK expression in *Gossypium hirsutum* is sensitive to, and generally downregulated by heat, cold, salt or drought (Qanmber et al., 2019). An exposition to a moderately low temperature leads to an expression change of a rice PERK gene, *Z15* (Feng et al., 2019). These findings collectively underscore the functional versatility of the *PERK* gene family in regulating fundamental plant life processes.

Advances in genome sequencing technologies have enabled systematic identification of *PERK* gene families across multiple plant species. While fifteen members have been confirmed in Arabidopsis, thirty-three were identified in cotton (*G. hirsutum*) (Qanmber et al., 2019). Cultivated peanut (*A. hypogaea* L.), an allopolyploid leguminous crop valued for its high oil and protein content, is predominantly cultivated in tropical and subtropical regions (Jiang et al., 2021). Current genomic evidence supports an evolutionary origin involving hybridization between two diploid progenitors (*Arachis duranensis* and *Arachis ipaensis*) followed by chromosomal duplication (Seijo et al., 2007). Breeding disease-resistant, stress-tolerant, and high-quality varieties represents a critical strategy to mitigate production constraints. Systematic investigations into the role of the *PERK* gene family in disease and stress resistance within peanut remain notably absent. As an allotetraploid species, peanut has undergone complex genome evolution, typically resulting in larger gene family sizes compared to diploid model plants (Huang et al., 2022). Therefore, comprehensive identification of peanut *PERK* family members and elucidation of their evolutionary relationships and expression dynamics are crucial prerequisites for uncovering the molecular mechanisms underpinning stress tolerance. Concurrently, the functional association between the *PERK* gene family and disease resistance pathways remains poorly characterized in peanut.

This study employs the peanut cultivar ‘Fuhuasheng’ to systematically identify and characterize the PERK gene family in peanuts. We aim to investigate the evolutionary features, expression patterns, and potential functional roles of these genes in development and stress responses. Furthermore, we seek to explore their involvement in the regulation of plant immunity. The findings are expected to provide insights into the functional diversification of PERK genes and offer potential genetic targets for stress-resistance breeding in legume crops.

## 2 Materials and methods

### 2.1 Plant materials

The peanut cultivar ‘Fuhuasheng’, utilized throughout this study, has undergone comprehensive whole-genome assembly and sequencing. Its publicly available reference genome database (Peanut Genome Resource, PGR: https://pgr.itps.ncku.edu.tw) served as the primary resource for in silico analyses. For experimental studies, ‘Fuhuasheng’ plants were cultivated in a growth chamber under a 14-h light/10-h dark (25 °C) photoperiod, with a light intensity of 350 µmol m⁻² s⁻¹ and 60-70% relative humidity. Plants were grown in a standard peat-based potting mix.

*Nicotiana benthamiana* and *A. thaliana* (Col-0) plants were maintained in a controlled-environment growth room at 22 °C with 70% relative humidity and a 14-h light/10-h dark photoperiod. A light intensity of 120 µmol m⁻² s⁻¹ was provided by cool-white fluorescent lamps. *N. benthamiana* plants were grown in a commercial potting soil mixture and used for transient expression assays at the 4–5 leaf stage. Arabidopsis plants grow on a 1:1 mixture of peat moss and vermiculite, and are used for Agrobacterium-mediated genetic transformation after flowering.

### 2.2 Identification of *PERK* family members

The complete genome sequence, annotation files, and gene structure information (introns, exons, coding sequences - CDS) for peanut were retrieved from the public PGR database. Full-length protein sequences corresponding to the 15 known *Arabidopsis thaliana PERK* genes (*AtPERKs*) served as query sequences for BLASTP searches against the peanut genome protein annotation dataset (Altschul et al., 1997). Structural characteristics of Arabidopsis PERK family are provided in **Supplementary Table S1**. Following removal of redundant sequences, candidate *PERK* homologs were validated using the SMART database to confirm the presence of characteristic PERK domain architecture prior to subsequent analyses (Letunic and Bork, 2018).

### 2.3 Sequence analysis and chromosomal localization of *PERKs*

Chromosomal positions and protein lengths of identified *PERK* genes were extracted directly from the PGR database annotations. Key physicochemical parameters of the deduced *PERK* proteins, including molecular weight and theoretical isoelectric point (pI), were calculated using the ExPASy Compute pI/Mw tool (https://web.expasy.org/compute_pi/; accessed on 20 June 2025). Subcellular localization predictions were generated using the DeepLoc-2.1 server (https://services.healthtech.dtu.dk/services/DeepLoc-2.1/; accessed on 23 December 2025).

### 2.4 Phylogenetic analysis and classification of *PERK*s

Multiple sequence alignment of the full-length amino acid sequences derived from the 23 identified AhPERKs and the 15 AtPERKs was performed using ClustalW (https://www.genome.jp/tools-bin/clustalw; accessed on 1 July 2025) (Thompson et al., 1994). A maximum-likelihood (ML) phylogenetic tree was constructed using MEGA 10.2 software (Kumar et al., 2018), with branch support assessed by 1,000 bootstrap replicates.

### 2.5 Analysis of *PERK* structural features

Conserved protein motifs within the *PERK* sequences were identified using the MEME suite (v5.3.3; https://meme-suite.org/meme/tools/meme; accessed on 1 July 2025) (Bailey et al., 2009) employing default search parameters. Exon-intron structural organization information for each *PERK* gene was extracted directly from the PGR database annotations.

### 2.6 Analysis of *Cis*-acting elements in *PERK* gene promoters

To investigate potential regulatory elements, a 2.0-kb genomic region immediately upstream of the translation initiation codon (ATG) for each PERK gene was extracted utilizing TBtools software (Chen et al., 2020). Putative *cis*-acting elements within these promoter sequences were identified and functionally annotated using the PlantCARE online platform (http://bioinformatics.psb.ugent.be/webtools/plantcare/html/; accessed on 5 July 2025) (Lescot et al., 2002).

### 2.7 Analysis of *PERK* gene expression patterns

Publicly available transcriptome datasets hosted within the PGR database were analyzed to determine *PERK* gene expression patterns across diverse tissues and under various stress conditions. Tissue samples analyzed included: Root, Root tip, Root nodule, Root and stem, Stem, Stem tip, Cotyledon, Leaf, Florescence (full flowering), Gynophore, Pericarp-I, Pericarp-II, Pericarp-III, Embryo-I, Embryo-II, Embryo-III, Embryo-IV, Testa-I, and Testa-II.

Stress treatments comprised: drought stress (normally irrigated leaves vs. drought-treated leaves), hormone treatments (ddH_2_O-, salicylic acid (SA)-, paclobutrazol (PAC)-, ethephon (ETH)-, brassinolide (BL)-, or abscisic acid (ABA)-treated leaves), and temperature stress (room temperature control leaves vs. low-temperature-treated leaves).

### 2.8 ROS detection assay

Full-length coding sequences of selected *PERK* genes were amplified via PCR and subsequently cloned into the pCAMBIA1300-35S-HA-RBS expression vector for Agrobacterium tumefaciens (strain GV3101)-mediated transient expression in mature leaves of 5- to 6-week-old *N. benthamiana* plants. Forty-eight hours post-infiltration, leaf discs (8 mm diameter) were excised using a sterile cork borer (Sigma-Aldrich) and equilibrated overnight in 200 μL of ultrapure water within 96-well plates. Discs were subsequently challenged with 200 μL of reaction buffer containing 20 μM L-012 (Wako Chemicals), 10 μg/mL horseradish peroxidase (HRP; Sigma-Aldrich), and 1 μM flg22 peptide. Luminescent signals generated by ROS production were quantified at 30-second intervals for a duration of 15 minutes using a Tecan F200 microplate reader. Three independent biological replicates were analyzed per construct, with statistical significance determined by one-way ANOVA followed by Tukey’s *post hoc* test (*p* < 0.05).

### 2.9 Pathogen infection assays

In the *Pst* DC3000 infection experiments, Arabidopsis plants cultivated in soil for 4–5 weeks were used. Bacterial suspensions at a concentration of 5 × 10^8^ CFU/mL were sprayed onto the leaves. Bacterial titers were assessed 3 days after inoculation.

### 2.10 RNA extraction and quantitative RT-PCR

Total RNA was extracted from Arabidopsis tissues employing the Plant Total RNA Kit (ZomanBio, Beijing, China). Subsequently, first-strand cDNA was reverse-transcribed using PrimeScript RT Master Mix (TaKaRa, Dalian, China). Quantitative real-time PCR (qRT-PCR) was carried out with SYBR^®^ Green Premix Pro Taq HS qPCR Kit (Accurate, Changsha, China) on an ABI QuantStudio 6 Flex instrument. All primer sequences utilized in this study are provided in **Supplementary Table S2**.

## 3 Results

### 3.1 Identification of PERK subfamily members and analysis of protein physiochemical properties in peanut

Systematic interrogation of the peanut genome annotation file using the BLASTP algorithm led to the identification of 23 *PERK* family members. These were designated *PERK1* to *PERK23* based on their sequential chromosomal locations (**Table 1**). Comprehensive characterization of these 23 *PERK* genes was performed, encompassing amino acid sequence length, predicted protein molecular weight (MW), theoretical isoelectric point (pI), and subcellular localization (**Table 1**). Results revealed moderate diversity in protein length, ranging from 503 amino acids (*PERK14*) to 761 amino acids (*PERK19*). Corresponding molecular masses varied from 55.18 kDa (*PERK14*) to 79.24 kDa (*PERK16*). Theoretical pI values spanned a broad range from 5.46 (*PERK21*) to 9.34 (*PERK5*), suggesting functional diversity. Subcellular localization predictions unanimously indicated cell membrane and/or lysosome/vacuole distribution for all PERK proteins. Detailed characteristics of the 23 *PERK* gene family members are compiled in **Table 1**.

**Table 1:** Characterization of 23 *PERK* gene family members in peanut.

Gene name	Gene id	Chromosome	Gene location	Protein length (aa)	Molecular weight (kDa)	Theoretical pl	Subcellular location
PERK1	AH03G40590	Chr03	133588184-133593047	699	75.16	7.07	Nucleus.
PERK2	AH04G28880	Chr04	121021572-121026057	512	55.95	8.95	Nucleus.
PERK3	AH05G06780	Chr05	8648102-8650787	625	67.68	8.06	Nucleus.
PERK4	AH05G06800	Chr05	8670000-8673385	628	66.16	5.88	Nucleus.
PERK5	AH05G12490	Chr05	31683123-31687737	666	72.12	9.34	Nucleus.
PERK6	AH05G26380	Chr05	95973043-95977000	672	70.88	8.61	Nucleus.
PERK7	AH05G33440	Chr05	108994047-108999993	748	77.78	8.61	Nucleus.
PERK8	AH07G19070	Chr07	58096312-58099405	674	70.81	5.47	Nucleus.
PERK9	AH09G02550	Chr09	2789404-2794566	574	61.47	7.28	Nucleus.
PERK10	AH10G21920	Chr10	99101774-99106185	666	70.60	5.55	Nucleus.
PERK11	AH10G21930	Chr10	99121004-99125847	697	74.06	5.84	Nucleus.
PERK12	AH13G43490	Chr13	134437744-134442248	698	75.15	6.88	Nucleus.
PERK13	AH14G33760	Chr14	123126844-123133143	509	55.82	8.46	Nucleus.
PERK14	AH14G33770	Chr14	123157796-123164857	503	55.18	6.90	Nucleus.
PERK15	AH14G37820	Chr14	128116988-128122185	756	79.15	8.22	Nucleus.
PERK16	AH14G42590	Chr14	132194628-132199717	756	79.24	8.50	Nucleus.
PERK17	AH15G02890	Chr15	4741895-4745514	625	65.92	5.78	Nucleus.
PERK18	AH15G10390	Chr15	19046161-19050561	665	72.18	9.30	Nucleus.
PERK19	AH15G18940	Chr15	107738643-107744435	761	79.03	8.75	Nucleus.
PERK20	AH15G29070	Chr15	141401608-141405505	672	70.88	8.61	Nucleus.
PERK21	AH17G19080	Chr17	60342854-60348362	680	71.64	5.46	Nucleus.
PERK22	AH19G03940	Chr19	3804743-3809866	574	61.46	7.30	Nucleus.
PERK23	AH20G28680	Chr20	126343458-126348165	693	73.68	5.92	Nucleus.

### 3.2 Chromosomal distribution of *PERK* subfamily members in peanut

**Figure 1:**
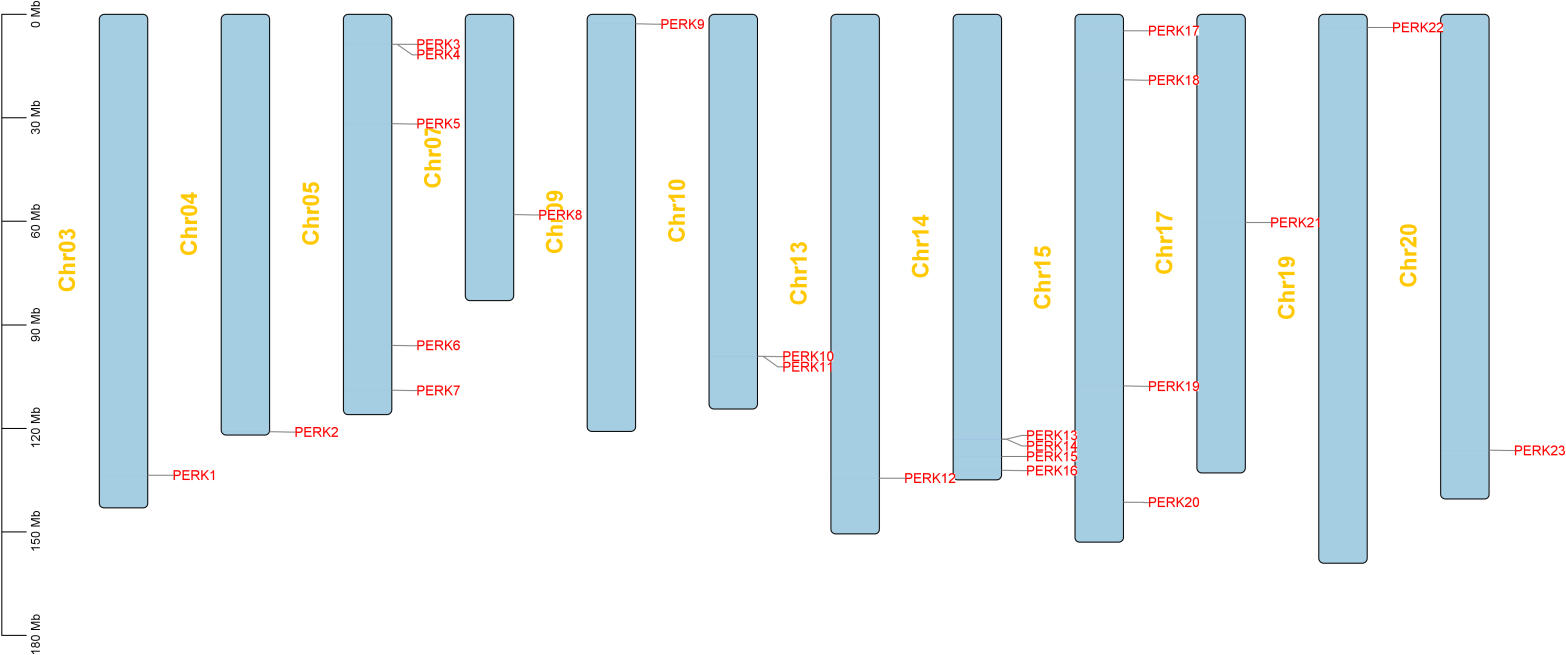
Chromosomal localization of *PERK* gene family members in peanut. 23 members were mapped to 12 chromosomes.

Chromosomal positional information for the 23 *PERK* genes was retrieved from the PGR database (**Table 1**). Localization analysis demonstrated that these genes are widely, yet unevenly, distributed across the peanut genome. They are located on 12 specific chromosomes: Chr03, Chr04, Chr05, Chr07, Chr09, Chr10, Chr13, Chr14, Chr15, Chr17, Chr19, and Chr20. The distribution density among chromosomes was highly asymmetric. Chromosome 5 harbored the highest number of *PERK* genes (5 genes), indicative of a region of elevated density. Chromosomes 14 and 15 each contained 4 *PERK* genes. Chromosome 10 possessed 2 *PERK* genes. The remaining chromosomes (Chr03, Chr04, Chr07, Chr09, Chr13, Chr17, Chr19, Chr20) each contained only a single *PERK* gene, signifying regions of sparse *PERK* gene distribution (**Figure 1**).

### 3.3 Phylogenetic analysis of the peanut *PERK* family

**Figure 2:**
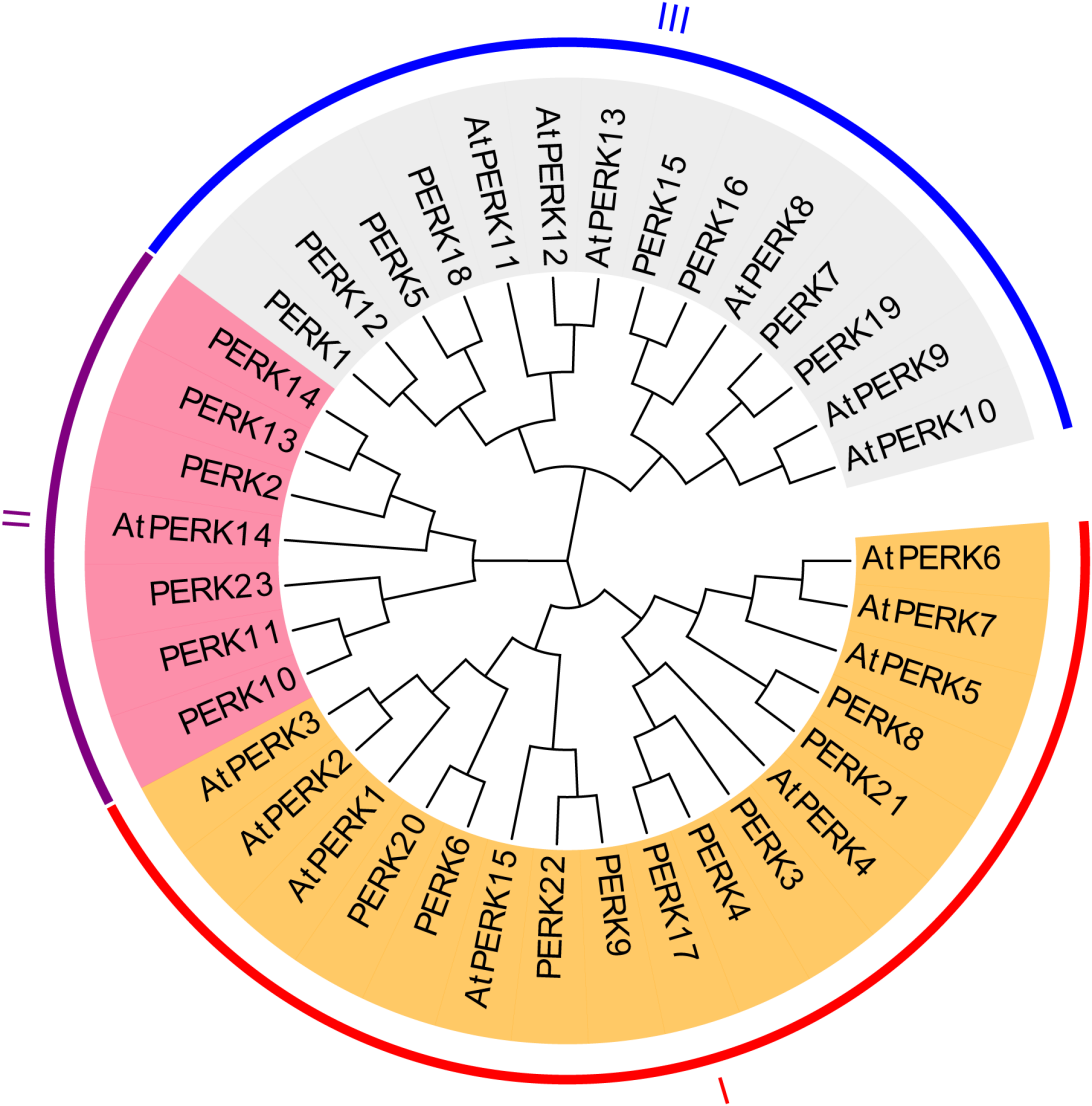
Phylogenetic tree of *PERK* proteins from *Arabidopsis* and peanut. A Maximum-likelihood phylogenetic tree illustrates evolutionary divergence among 15 *Arabidopsis* and 23 peanut PERK homologs. Full-length protein sequences were aligned using ClustalW, and the tree was constructed with 1000 bootstrap replicates using MEGA X.

To elucidate the evolutionary relationships between peanut and Arabidopsis *PERK* genes, a maximum likelihood (ML) phylogenetic tree was constructed based on multiple sequence alignments of the full-length amino acid sequences of the 23 peanut *PERK* proteins and 15 Arabidopsis *PERK* proteins. Based on sequence similarity and phylogenetic topology, the peanut *PERK* genes were resolved into three primary clades, designated Group I, Group II, and Group III. As depicted in **Figure 2**, Group I comprised 9 peanut *PERK* proteins and 8 Arabidopsis *PERK* proteins. Group II contained 6 peanut *PERK* proteins and 1 Arabidopsis *PERK* protein. Group III included 8 peanut *PERK* proteins and 6 *Arabidopsis PERK* proteins (**Figure 2**).

### 3.4 Analysis of gene structure and conserved protein motif composition in the peanut *PERK* gene family

**Figure 3:**
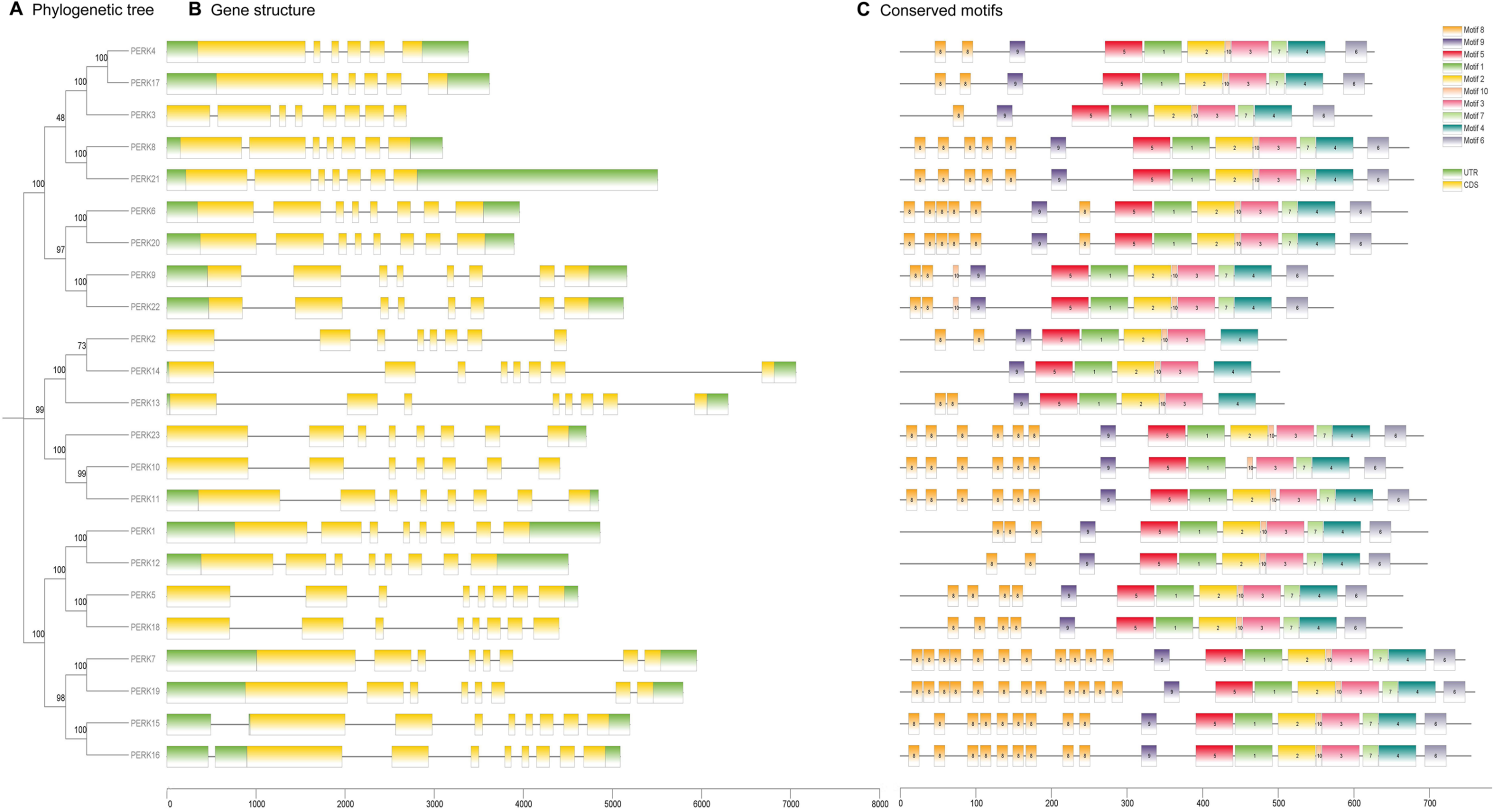
Evolutionary relationships **(A)**, gene structure **(B)**, and conserved motifs **(C)** of *PERK* family members in Peanut. **(A)** Maximum likelihood phylogenetic tree of 23 PERK proteins, classified into three distinct clades using MEGA X. **(B)** Exon-intron architecture of *PERK* genes. Untranslated regions (UTR) are depicted as green boxes, coding sequences (CDS) as yellow boxes, and introns as gray connecting lines. **(C)** Conserved protein motif composition identified by MEME suite analysis. Ten motifs (Motif 1–Motif 10) are color-coded to reflect their sequential positions in protein sequences.

To gain insights into the evolutionary dynamics of the *PERK* gene family in peanut, the exon-intron structures were analyzed in conjunction with a phylogenetic tree constructed from the full-length *PERK* protein sequences. As shown in **Figure 3A**, the 23 peanut *PERK* genes were phylogenetically classified into three distinct subgroups. Gene structure analysis confirmed that all *PERK* gene family members in peanut contain introns (**Figure 3B**). The function and structure of *PERK* proteins are critically dependent on conserved motifs, which play essential roles in protein activity. In-depth motif analysis using the MEME suite identified 10 conserved motifs within the peanut *PERK* family (**Figure 3C**). All members possess Motifs 1, 3, 4, 5, 9, and 10. All members except *PERK10* contain one instance of Motif 2. Motifs 6 and 7 are absent in *PERK2, PERK13*, and *PERK14* but are present once in all other members. Within the peanut PERK family, all members except *PERK14* contain at least one instance of Motif 8. Notably, the copy number of Motif 8 exhibited the greatest variation across family members, suggesting its potential contribution to functional diversification within the family (**Figure 3C**). The detailed sequences of the six putative motifs are shown in **Supplementary Table S3**.

### 3.5 Analysis of stress-related *Cis*-acting elements in the promoters of peanut *PERK* genes

**Figure 4:**
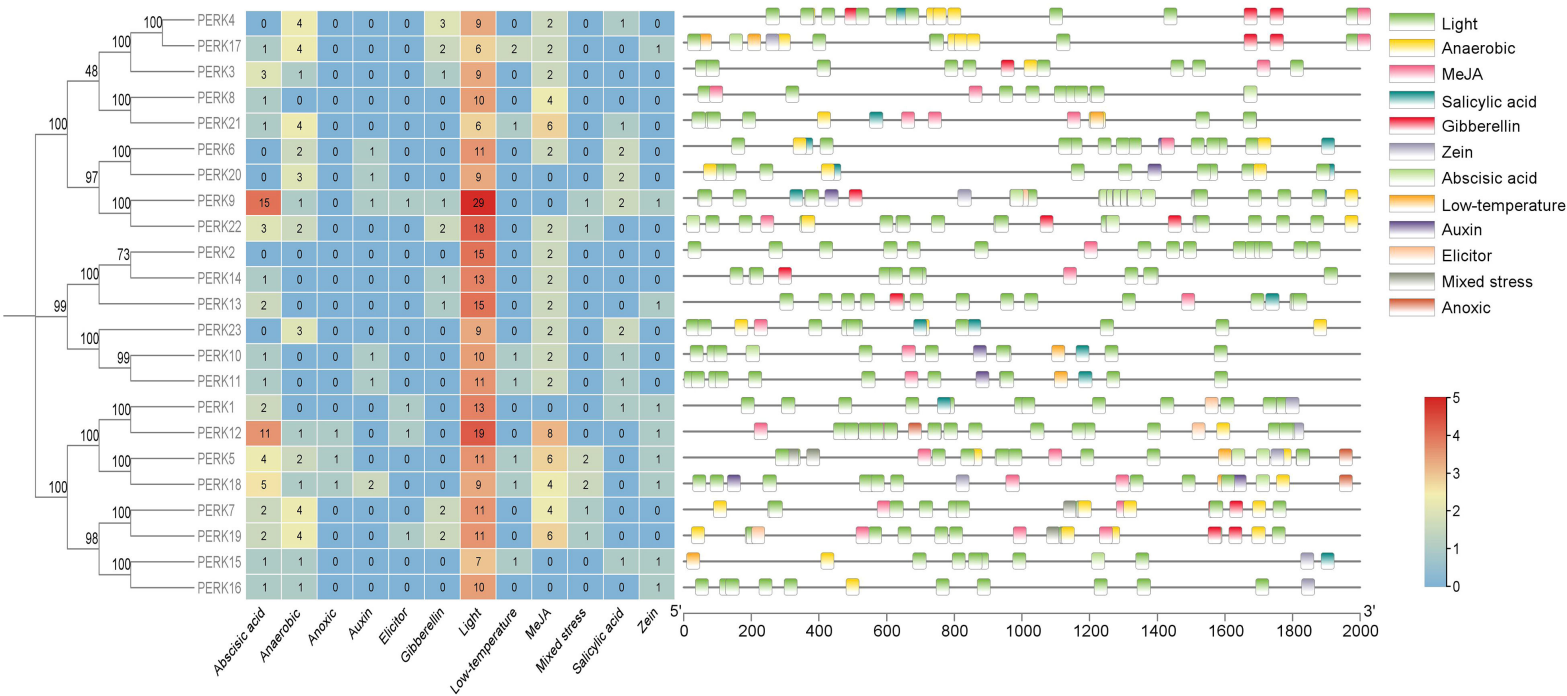
Distribution of *cis*-acting elements in the promoters of *PERK* genes in peanut. Schematic representation of stress- and hormone-responsive elements relative to the translation start site (TSS, position 0). Element positions were calculated using PlantCARE database annotations, with color-coded boxes denoting distinct regulatory modules.

To investigate the potential molecular mechanisms governing peanut *PERK* gene responses to abiotic stress and phytohormone signaling, a 2.0 kb genomic region upstream of the initiation codon (ATG) for each *PERK* gene was extracted from the PGR database. These promoter sequences were analyzed using the PlantCARE platform for the identification of stress-associated *cis*-acting regulatory elements. Analysis identified 12 distinct types of responsive *cis*-acting elements within the promoters of the 23 peanut PERK genes (**Figure 4**), encompassing elements associated with ABA response, anaerobic response, mixed stress response, zein response, light response, low-temperature response, MeJA response, salicylic acid (SA) response, auxin response, gibberellin (GA) response, elicitor response, and anoxic response.

As illustrated in **Figure 4**, results demonstrate that all 23 peanut *PERK* genes harbor at least 2 (*PERK2*) stress-responsive *cis*-acting elements, strongly suggesting their functional involvement in stress response. Association with light-responsive elements varied considerably among *PERK* members, ranging from a minimum of 6 elements (*PERK21*) to a maximum of 29 elements (*PERK9*). Critically, all *PERK* genes possess light-responsive *cis*-acting elements, representing the most abundant category identified. Furthermore, within the *PERK* family, only *PERK5*, *PERK12*, and *PERK18* contain anoxic response elements, which constitute the least frequent stress response element type. Similarly, only four genes *(PERK1, PERK9, PERK12*, and *PERK19*) each harbor a single elicitor response-related *cis*-acting element (**Figure 4**).

Within the *PERK* gene family, 18 members contain ABA-responsive *cis*-acting elements, and 16 members contain anaerobic response-related elements. These elements are likely instrumental in regulating *PERK* gene expression during ABA signaling and anaerobic responses. *Cis*-acting elements associated with auxin response, zein metabolism response, low temperature response, SA response, MeJA response, and mixed stress response were identified in 6, 9, 7, 10, 18, and 6 *PERK* genes, respectively. Nine *PERK* genes contain one or more GA-responsive *cis*-acting elements (**Figure 4**). Collectively, the *cis*-element analysis strongly suggests potential involvement of all 23 *PERK* genes in mediating responses to diverse environmental stresses and hormonal cues.

### 3.6 Expression pattern analysis of peanut *PERK* genes in different tissues

**Figure 5:**
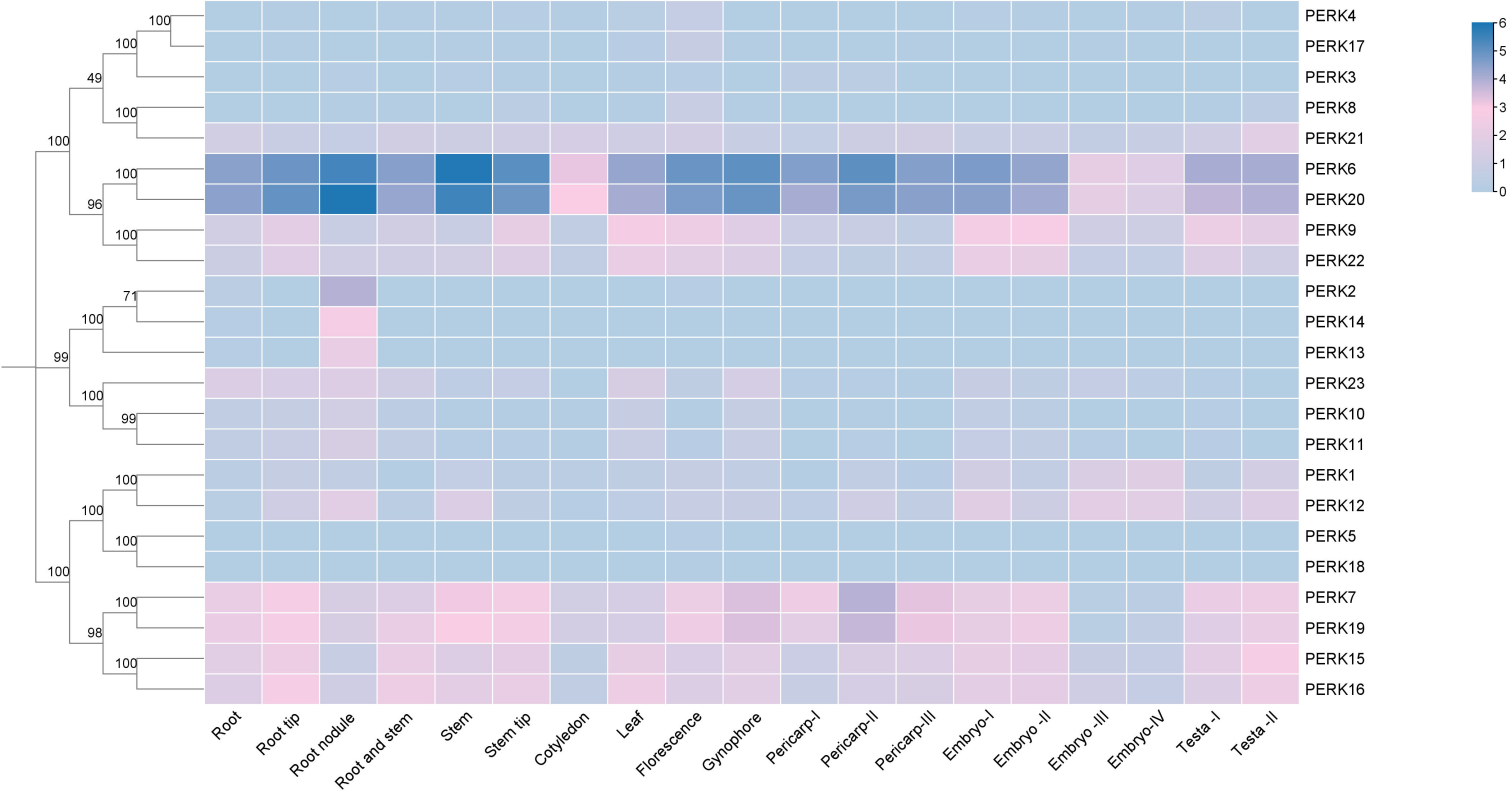
Heat map showing the expression levels of *PERK* genes in various tissues of peanut. Tissue-specific expression profiles of *PERK* genes based on RNA-seq data. Heatmap visualization represents log2-transformed FPKM values averaged across biological replicates. Color gradients indicate low-to-high expression levels, with hierarchical clustering revealing organ-specific expression patterns.

Genes typically exhibit distinct expression profiles across tissues or organs, reflecting their roles in regulating specific physiological processes. To elucidate the tissue-specific expression patterns of the peanut *PERK* gene family, transcript abundance (FPKM values) was analyzed across diverse tissues: Root, Root tip, Root nodule, Root and stem, Stem, Stem tip, Cotyledon, Leaf, Florescence, Gynophore, Pericarp-I, Pericarp-II, Pericarp-III, Embryo-I, Embryo-II, Embryo-III, Embryo-IV, Testa-I, and Testa-II. Transcript abundance data for the 23 peanut *PERK* genes across these tissues are presented in **Figure 5**.

All 23 *PERK* genes were expressed in at least one examined tissue. Analysis revealed that 11 *PERK* genes exhibited ubiquitous expression across all tissues analyzed. No family member demonstrated universally high expression (FPKM values > 3). Genes such as *PERK6* and *PERK20* displayed high expression levels (FPKM values > 3) in the majority of tissues. *PERK2, PERK13, PERK14*, and *PERK18* exhibited highly tissue-specific expression profiles: *PERK2* expression was restricted to Root, Root nodule, and Florescence; *PERK13* and *PERK14* were exclusively expressed in Root and Root nodule; *PERK18* was solely detected in Leaf and Florescence (**Figure 5**). This pronounced tissue specificity suggests potential specialized roles for these genes in regulating functions unique to these organs.

### 3.7 Expression patterns of *PERK* genes in response to phytohormone and abiotic stress treatments

To elucidate the functional roles of peanut *PERK* genes under phytohormone application and abiotic stress conditions, the transcriptional responses of the 23 *PERK* genes were systematically analyzed following treatments with SA, ethephon, BR, ABA, paclobutrazol, as well as exposure to drought, ddH_2_O control, and low temperature.

**Figure 6:**
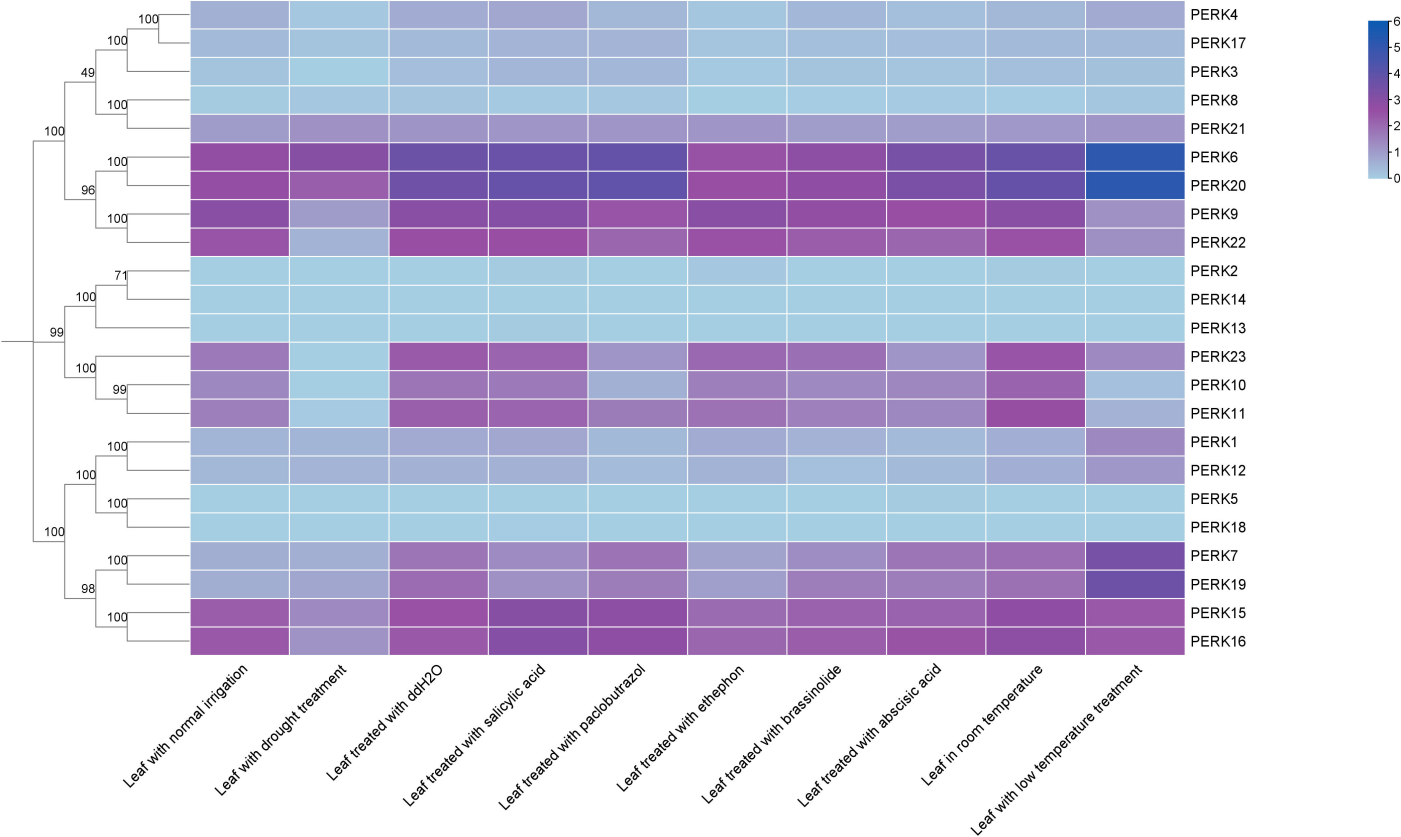
Expression patterns of peanut *PERK* genes in response to phytohormones and abiotic stresses. Heatmap analysis of log2(FPKM) values demonstrates transcriptional responsiveness to phytohormones and abiotic stresses.

Transcript abundance data for the 23 *PERK* genes are presented in **Figure 6**. Results showed that SA treatment significantly altered the expression of 2 genes (*PERK8* and *PERK19*), defined as a fold change ≥ 2. Paclobutrazol treatment significantly altered the expression of 3 genes (*PERK1*, *PERK10*, and *PERK23*). Ethephon treatment induced significant changes in *PERK3*, *PERK4*, *PERK6*, *PERK7*, *PERK17*, *PERK19*, and *PERK20* expression. BR treatment significantly altered the expression of 5 genes (*PERK6, PERK8, PERK11, PERK12*, and *PERK20*). Under ABA treatment, 6 genes (*PERK1, PERK3, PERK4, PERK8, PERK11*, and *PERK23*) exhibited significant expression level fluctuations. Drought stress significantly altered the expression of 9 genes (*PERK4, PERK8, PERK9, PERK11, PERK15, PERK16, PERK17, PERK22*, and *PERK23*), highlighting their important roles in drought response. In contrast, low temperature treatment significantly altered the expression of 12 genes: *PERK1, PERK6, PERK7, PERK8, PERK9, PERK10, PERK11, PERK12, PERK19, PERK20, PERK22*, and *PERK23* (**Figure 6**).

### 3.8 Analysis of *PERK* genes in flg22-induced ROS production

**Figure 7:**
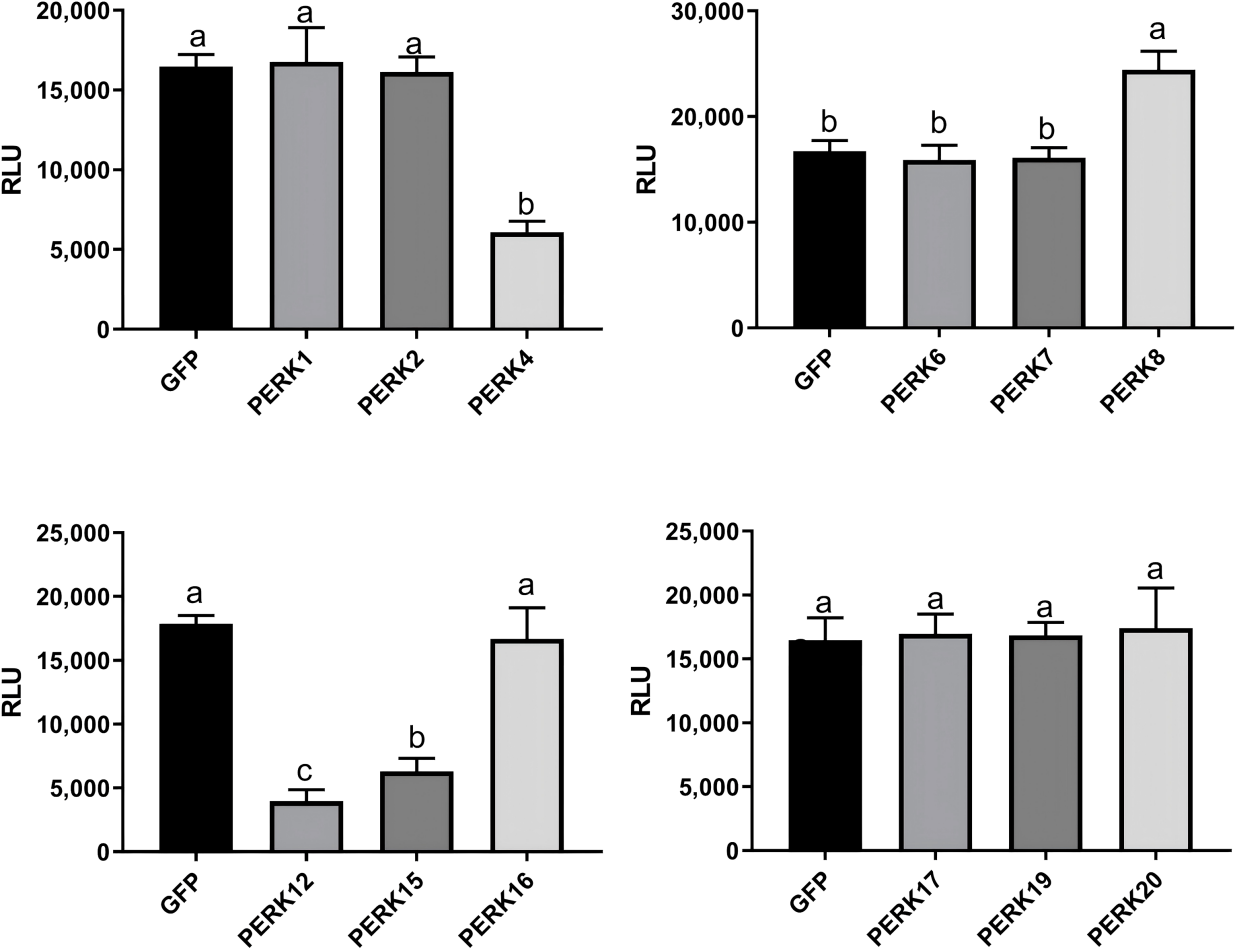
The Modulation of PAMP-triggered ROS by PERKs. The indicated constructs were transiently expressed by agrobacterium-mediated transient expression for 2 days and subjected to flg22-induced ROS examination (mean ± SD, n ≥ 8, and one-way ANOVA followed by Tukey’s *post hoc* test; different letters indicate significant difference at *p* < 0.01).

To further investigate the functional roles of *PERK* genes in plant immunity, 12 *PERK* family members were successfully cloned. Their regulatory activity in flg22-induced ROS production was assessed using a transient expression assay in *N. benthamiana*. We observed that the expression of 3 genes (*PERK4*, *PERK12*, *PERK15*) strongly suppressed flg22-induced ROS accumulation, while the expression of 1 gene (*PERK8*) strongly enhanced flg22-induced ROS burst (**Figure 7**). This functional divergence suggests distinct roles for these *PERK* members in either suppressing or enhancing plant basal immune responses during pathogen perception.

### 3.9 *PERK* proteins exhibit bidirectional regulatory roles in arabidopsis immunity

To further investigate the roles of *PERK4*, *PERK12*, *PERK15*, and *PERK8* in plant immunity, we introduced *PERK4-FLAG*, *PERK12-FLAG*, *PERK15-FLAG*, and *PERK8-FLAG* into Arabidopsis via *Agrobacterium*-mediated stable transformation and obtained two independent transgenic lines for each construct: *PERK4-FLAG-L1*, *PERK4-FLAG-L2*, *PERK12-FLAG-L1*, *PERK12-FLAG-L2*, *PERK15-FLAG-L1*, *PERK15-FLAG-L2*, *PERK8-FLAG-L1*, and *PERK8-FLAG-L2*.

**Figure 8:**
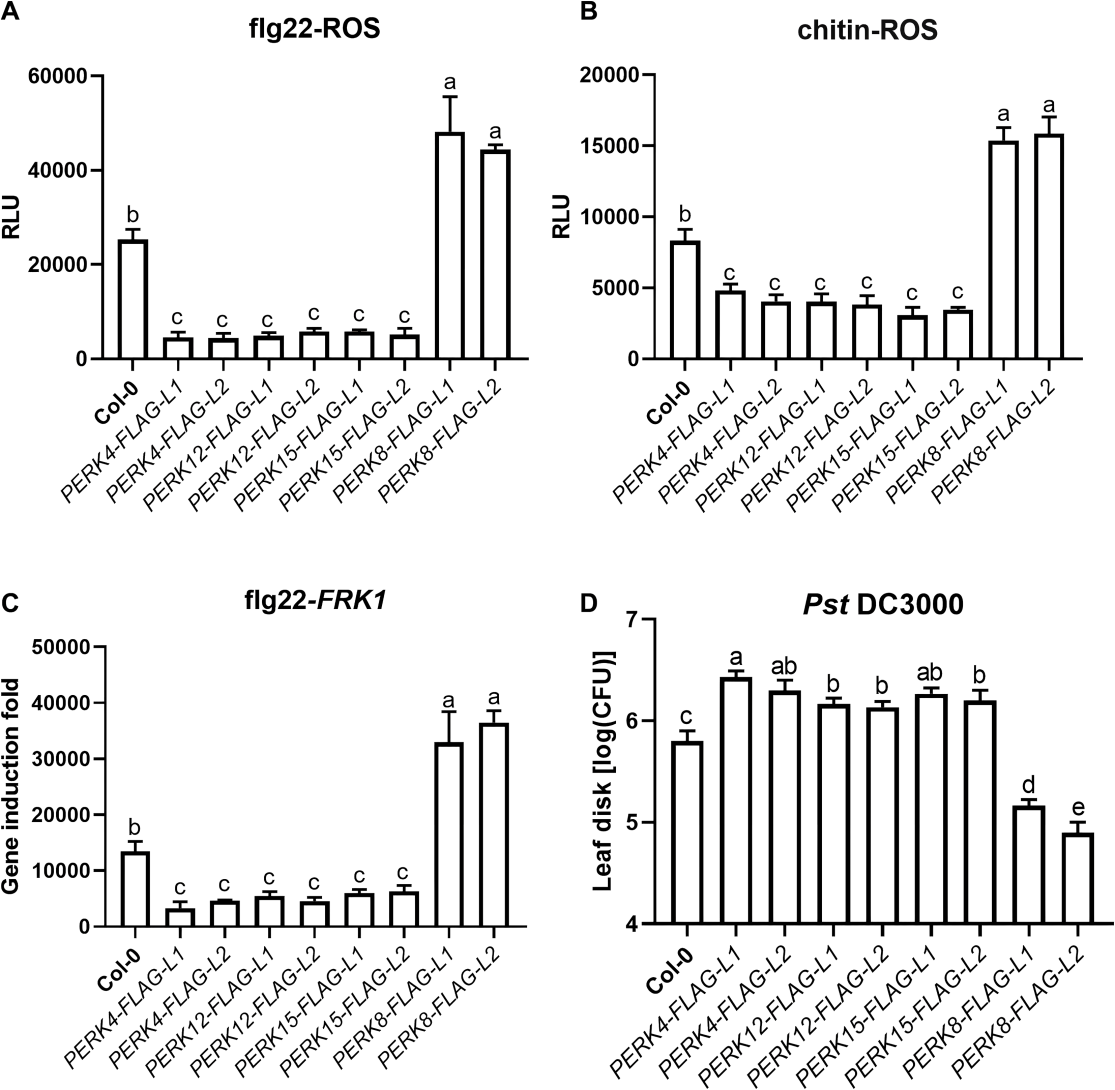
PERK proteins exhibit bidirectional regulatory roles in arabidopsis immunity. **(A)** flg22-induced ROS burst in leaves of Col-0 and the indicated *PERK-FLAG* transgenic lines. **(B)** chitin-induced ROS burst in the same set of plants as in **(A)**. **(C)** Relative expression levels of the defense marker gene *FRK1* in Col-0 and transgenic lines after flg22 treatment, measured by qRT-PCR. **(D)** Bacterial growth of *Pst* DC3000 in leaves of Col-0 and transgenic lines. Data represent mean ± SD from biologically independent replicates. Different lowercase letters above the bars indicate statistically significant differences as determined by one-way ANOVA followed by Tukey’s test (*p* < 0.05).

We subsequently examined PAMP-induced ROS production, PR gene expression, and resistance to *Pst* DC3000 in these transgenic lines. Our results showed that flg22-induced ROS burst was significantly damaged in the *PERK4*-, *PERK12*-, and *PERK15*-related transgenic plants compared to Col-0, whereas it was markedly enhanced in *PERK8* transgenic lines. This indicates that stable expression of PERK4, PERK12, and PERK15 proteins significantly suppresses flg22-induced ROS burst, while stable expression of PERK8 enhances it (**Figure 8A**). We also detected chitin-induced ROS production in these transgenic lines, and the results were consistent with the trends observed for flg22-induced ROS burst (**Figure 8B**).

Consistent with these findings, we further observed that flg22-induced expression of *FRK1*, a commonly used defense marker gene, was significantly reduced in the *PERK4*-, *PERK12*-, and *PERK15*-related transgenic plants. In contrast, flg22-induced *FRK1* expression was slightly enhanced in the *PERK8*-related transgenic lines (**Figure 8C**).

To assess the roles of PERKs in plant disease resistance, we challenged the transgenic plants with *Pst* DC3000. Compared with Col-0 plants, the *PERK4*-, *PERK12*-, and *PERK15*-related transgenic lines exhibited significantly enhanced susceptibility to *Pst* DC3000. Conversely, the *PERK8*-related transgenic plants showed markedly enhanced resistance to *Pst* DC3000 (**Figure 8D**).

Taken together, these results indicate that *PERK4*, *PERK12*, and *PERK15* negatively regulate plant basal immune responses, whereas *PERK8* acts as a positive regulator, revealing their bidirectional immune regulatory functions.

## 4 Discussion

This study provides the first systematic identification of 23 *PERK* gene family members within the peanut genome, coupled with a comprehensive analysis of their evolutionary characteristics, expression dynamics, and functional implications. As an important subfamily of RLKs, *PERK* genes are established as pivotal regulators of plant growth, development, and stress adaptation (Morris and Walker, 2003). We observed that the number of peanut *PERK* genes (23) is intermediate to that of *A. thaliana* (15) (Silva and Goring, 2002) and the allotetraploid cotton (33) (Qanmber et al., 2019), suggesting that the expansion of the *PERK* family in legumes correlates with genomic polyploidization levels. Notably, all identified peanut *PERK* genes contain introns (**Figure 3B**), contrasting sharply with the complete lack of introns reported for cotton *GhPERK* genes (Qanmber et al., 2019). This fundamental difference likely reflects distinct evolutionary trajectories and selective pressures. Variations in exon-intron structures typically arise from insertion/deletion events, and the consistent retention of introns in peanut *PERK*s suggests this family may occupy a relatively conserved evolutionary niche within legumes (Roy and Gilbert, 2005).

Phylogenetic classification grouped AhPERKs into three clades orthologous to those in Arabidopsis (**Figure 2**). Notably, Group II experienced a significant expansion in peanut, containing six members versus only one in Arabidopsis. This lineage-specific expansion hints at functional diversification or neofunctionalization in legumes, potentially linked to their unique biological traits like symbiotic nitrogen fixation. Supporting this, our promoter analysis revealed a rich repertoire of *cis*-elements associated with light response, ABA, and MeJA, underscoring the potential involvement of AhPERKs in integrating environmental and hormonal signals—a hallmark of RLK function (Invernizzi et al., 2022). These results align with the observed MeJA/ABA responsiveness of GhPERKs in cotton (Qanmber et al., 2019) and studies demonstrating AtPERK4-mediated ABA signaling in *Arabidopsis* (Bai et al., 2009a, b), supporting the conserved function of the *PERK* family in hormone regulation.

Tissue expression profiles indicated functional divergence among peanut *PERK* genes: 11 genes exhibited constitutive expression, while *PERK2, PERK13, PERK14*, and *PERK18* displayed tissue-specific patterns (**Figure 5**). Of particular interest is the expression of *PERK13/PERK14* exclusively in roots and root nodules, implying potential roles in the legume-specific symbiotic nitrogen fixation process. This strongly suggests a previously unexplored role for PERKs in the legume-specific symbiosis with rhizobia. Given that PERKs are hypothesized to sense cell wall dynamics (Borassi et al., 2016; Invernizzi et al., 2022), their expression in nodules—organs undergoing extensive cell wall remodeling—positions them as prime candidates for regulating infection thread development, symbiosome formation, or nodule organogenesis. This discovery opens a new avenue for research into how cell wall-associated receptors modulate symbiotic efficiency. Under abiotic stress conditions, 12 genes were significantly induced by low temperature and nine responded to drought stress (**Figure 6**). *PERK1, PERK8, PERK11*, and *PERK23* were concurrently activated by both ABA and drought, consistent with the presence of predicted ABA-responsive elements in their promoters (**Figure 4**) and previous findings on *PERK*-mediated ABA signaling (Bai et al., 2009a).

The uneven chromosomal distribution of peanut *PERK* genes (**Figure 1**) may stem from local tandem duplication or chromosomal fragmentation events (Schauser et al., 2005), requiring validation via synteny analysis. Although gibberellin-responsive elements were predicted in promoters, GA-related data is absent from the phytohormone treatment experiments, necessitating supplementary validation. Future research should employ gene-editing technologies to generate *PERK* mutants for in-depth characterization of their molecular mechanisms in symbiotic nitrogen fixation (e.g., nodule development) and immune responses (e.g., ROS regulation).

Our study provides crucial functional insights into PERK-mediated immunity. While PERKs have been implicated in responses to pathogens and herbivores (Florentino et al., 2006; Invernizzi et al., 2022), their direct regulatory mechanisms in immune signaling remain opaque. Our functional assays using flg22-induced ROS burst as a readout for Pattern-Triggered Immunity (PTI) unveiled a bidirectional regulatory capacity among AhPERKs. Specifically, *AhPERK4*, *AhPERK12*, and *AhPERK15* acted as negative regulators, suppressing ROS accumulation, defense gene expression, and resistance to *Pseudomonas syringae*. Conversely, *AhPERK8* functioned as a positive regulator, enhancing these immune outputs. This divergence aligns with the emerging paradigm that RLK networks can finely tune immune responses through opposing actions (Zhou and Zhang, 2020). The negative regulation by some AhPERKs echoes the role of certain PERKs as negative modulators of growth processes like root elongation (Humphrey et al., 2015), suggesting a conserved function in signaling attenuation. The positive role of *AhPERK8* finds a parallel in the wound-induced *AtPERK1* (Silva and Goring, 2002), hinting at a potential conserved activation mechanism under different stresses. These results significantly advance current knowledge by moving beyond correlative expression studies to demonstrate direct and differential roles for PERK family members in modulating a key immune signaling pathway.

## 5 Conclusion

This study provides a comprehensive genomic and functional characterization of the PERK gene family in peanut. We identified 23 AhPERK genes, revealing their non-uniform chromosomal distribution, conserved gene structures, and diversification into three phylogenetic subgroups. Promoter analysis indicated their potential involvement in diverse stress responses, which was corroborated by expression profiling showing that specific AhPERK members are differentially regulated by abiotic stresses and hormone treatments. Crucially, tissue-specific expression patterns suggest roles in both fundamental development and specialized root/nodule functions. The most significant finding is the functional divergence of AhPERK members in plant immunity. Functional validation demonstrated that *AhPERK4*, *AhPERK12*, and *AhPERK15* act as negative regulators, while *AhPERK8* acts as a positive regulator of basal immune responses, including PAMP-triggered ROS burst, defense gene expression, and resistance to bacterial pathogens. This reveals, for the first time in peanut, the bidirectional regulatory capacity of the PERK family in plant immunity. Collectively, our results establish a foundation for understanding the multifaceted roles of AhPERK genes, linking their structural features to expression dynamics and critical immune functions. These findings highlight specific AhPERK genes as promising candidates for further mechanistic studies and as potential genetic targets for enhancing stress resilience in legume crops.

## Data Availability

The original contributions presented in the study are included in the article/**Supplementary Material**. Further inquiries can be directed to the corresponding author/s.
